# Functional Imaging of Craving

**Published:** 1999

**Authors:** Daniel W. Hommer

**Affiliations:** Daniel W. Hommer, M.D., is chief of the Section of Brain Electrophysiology and Imaging, Laboratory of Clinical Studies, Division of Intramural Clinical and Biological Research, at the National Institute on Alcohol Abuse and Alcoholism, Bethesda, Maryland

**Keywords:** AOD (alcohol and other drug) craving, single photon emission computed tomography, positron emission tomography, magnetic resonance imaging, brain imaging, blood flow measurement, cocaine, brain, basal ganglia, limbic system, brain function, neurotransmission, dopamine, glucose metabolism, literature review

## Abstract

To visualize brain activity associated with mental states, such as craving for alcohol and other drugs (AODs), researchers have begun to use functional imaging techniques. Three commonly used techniques are single photon emission computed tomography (SPECT), positron emission tomography (PET), and functional magnetic resonance imaging (fMRI). Studies using these three approaches have been reviewed in order to evaluate the validity of a proposed model of the brain regions involved in alcoholism and the craving for alcohol. This model suggests a central role for a connected group of brain regions that include the basal ganglia, thalamus, and orbital cortex. A study using SPECT technology in alcoholics, however, found altered brain activity in only some of those regions during craving. Additional studies in alcoholics, as well as cocaine users, identified several other brain regions whose activities appeared to change in response to craving. These studies have led to the development of a revised model of brain regions involved in craving for AODs. Numerous questions remain, however, that must be answered before the brain areas involved in craving can be identified conclusively.

Primates, including humans, are visual animals—that is, they like to see things—giving rise to the adage “seeing is believing.” One cannot see thoughts, feelings, or mental states, however, whether one’s own or those of other people. One can only perceive these states either directly, in one’s own consciousness, or indirectly, through the reports and behavior of other people. “Craving” is a term derived from popular psychology that is used to describe one of these mental states—namely, the intense desire for a certain object or experience (e.g., alcohol or other drugs [AODs]).

Cognitive neuroscience postulates that mental states are the product of neurochemical processes in specific brain regions (i.e., brain states). Although one cannot see mental states, one can generate images that help visualize the intensity and location of physiological processes associated with brain states.

At the most basic level, the physiological processes underlying brain states involve changes in and the maintenance of differences in the concentrations of molecules that carry electrical charges (i.e., ions) across the membrane forming the boundary of each nerve cell, or neuron. Changes in the concentrations of various ions inside and outside of the cell serve as signals that can be transmitted from one neuron to another. Because differences in ion concentrations already exist between the inside and the outside of a resting or inactive neuron, the process of moving additional ions to generate a nerve signal requires energy. In the brain, this energy is supplied by the brain’s metabolism of the sugar glucose, which is delivered to the brain through the bloodstream. Accordingly, when a certain brain region is actively involved in the generation of mental states, the energy requirements of that region increase and, consequently, so does the blood flow there. By measuring blood flow or glucose metabolism (a process known as functional brain imaging), one can determine which brain areas are most active during a particular brain state.

This article reviews the small number of studies that have applied functional brain imaging to investigating brain states associated with craving for AODs, particularly alcohol and cocaine. It includes studies on cocaine craving because (1) to date, only two studies have attempted to image craving for alcohol, and (2) the brain states associated with craving may be similar for all AODs. The article first describes the three most commonly used functional imaging techniques. It then introduces a preliminary model of the functional anatomy of craving to provide a framework for understanding the studies on craving conducted among alcoholics and cocaine abusers. After comparing the results of studies that examine the preliminary model, the article discusses future directions in the functional imaging of craving.

## Methods of Functional Brain Imaging

Currently three major techniques are used to visualize the brain activity associated with various mental states: single photon emission computed tomography (SPECT), positron emission tomography (PET), and functional magnetic resonance imaging (fMRI). Each technique involves measuring local changes in cerebral^1^ blood flow or metabolism. To that end, researchers usually obtain at least two separate images representing two different states of the brain regions of interest. In theory, one could just visually compare the two images to identify brain regions in which brain activity differs depending on mental state. In many cases, however, the differences between the two images are too subtle to be detected or quantified accurately by simple visual inspection. Therefore, a new image representing the differences between the two original images (i.e., a difference image) is created using computerized analysis. This can be done because each image is made up of small units, or pixels, each of which represents a small brain area. For each pixel the blood-flow or glucose-metabolism value in the first image is “subtracted” from the value in the corresponding pixel in the second image. For example, assume that the blood flow in a given pixel is low in the first brain state (e.g., no craving) and is assigned a value of 2 on a scale from 0 to 10. In the second brain state (e.g., craving), the blood flow in the same pixel is high (i.e., is assigned a value of 9). Thus, in the difference image, this pixel will have a blood flow value of 7, representing a relatively large difference between the two brain states. This subtraction process is performed for all pixels in the images, generating an image that represents the extent of changes in blood flow between the two brain states being assessed. Statistical analysis of several difference images then allows the identification of the brain regions whose activities vary most strongly between the two brain states.

The key to this approach is to select tasks that will induce brain states that differ only with regard to the mental state being investigated. In a study of alcohol craving, for example, investigators might have alcoholics watch two different videotapes: One video might show people drinking alcoholic beverages, whereas the other video might show people engaged in an activity unrelated to drinking. At least in theory, the only difference between the mental states evoked by the two videos would be that one video would induce a desire to drink, and the other video would not. Thus, the difference image produced in this experiment would represent the blood flow or glucose metabolism associated with alcohol craving. However, researchers cannot be absolutely certain that any differences between the brain states induced by the two videos would result only from the presence or absence of craving. Other influences not related to craving, such as differences between the two videos with regard to their visual properties (e.g., color and movement), also may affect the difference image. Consequently, it is important that investigators try to minimize differences between the stimuli used to evoke the desired brain states other than the one difference that they are attempting to visualize.

### Single Photon Emission Computed Tomography

SPECT is the oldest functional imaging technique and generally provides images with the lowest resolution, although newer SPECT equipment provides images with significantly greater detail. With this approach, the subject is injected with a weakly radioactive tracer—usually ^99m^technetium-hexamethylpropy-lene-amineoxide (^99m^Tc-HMPAO)—to measure blood flow. This technique allows the investigator to visualize the blood flow distribution within the brain during the first 1 or 2 minutes after ^99m^Tc-HMPAO injection. The major advantage of this approach is that it is more widely available and much less expensive than other imaging techniques, such as PET.

SPECT also has substantial disadvantages, however. The biggest disadvantage is that because ^99m^Tc-HMPAO decays slowly (i.e., has a long half-life), the two images needed to produce a difference image must be recorded several days apart, leading to two practical problems. First, each time a subject is placed in the machine (i.e., the scanner) that measures the radioactivity and generates the image, his or her positioning will be slightly different. This inconsistency complicates the generation of an accurate difference image, because the two original images must be aligned so that their size, shape, and orientation match perfectly. Second, mental states and the resulting brain states are likely to vary more when they are recorded a few days apart than when they are recorded a few minutes or hours apart. As a result of the long separation between scans required by SPECT, difference images can reflect changes in brain states merely associated with the passage of time rather than specifically with the mental state under investigation.

### Positron Emission Tomography

For PET, subjects are injected with molecules that naturally occur in the tissues, such as water (H_2_O) and glucose, and which have been tagged with a radioactive variant (i.e., isotope) of one of their atoms. These radioactive variants are taken up into the tissues just like the normal molecules, and their distribution can be measured using a scanner. The most commonly employed PET technique measures blood flow using radioactively labeled [^15^O] H_2_O. Each PET image represents the blood flow during the first 1 or 2 minutes following ^15^O administration. This approach offers several advantages over SPECT. For example, because ^15^O has a half-life of only a few minutes, six to eight images can be produced during one imaging session. This feature reduces imaging problems that result from the subject’s movement, incorrect alignment of the images, and day-to-day variations in mental state.

Another tracer used in PET imaging is radioactively labeled glucose (i.e., [^18^F]-fluorodeoxyglucose [FDG]). This compound, which is taken up by cells that are in the process of generating energy from glucose breakdown (i.e., metabolically active cells), is then trapped within the cells so that its location can be imaged. In contrast to ^15^O PET’s short measurement period, FDG PET measures neuronal glucose use during a period of 20 to 30 minutes. This extended measurement allows the generation of more accurate images compared with ^15^O PET. A disadvantage of the prolonged measurement period needed to generate one image, however, is that FDG PET allows the generation of only one pair of images, and thus of one difference image, per day.

A major advantage of PET over SPECT is that it generates images with a greater resolution: The decay of each radioactive isotope used in PET (i.e., ^15^O and ^18^F) eventually generates two photons that can be detected by a special camera. Conversely, the decay of each tracer molecule used in SPECT generates only one photon, thereby allowing less precise localization of the tracer molecules.

PET also is associated with two major disadvantages, however. First, because the ^15^O and ^18^F tracers have such short half-lives, they must be generated immediately before use—a labor-intensive and expensive process. Thus, a machine to generate these tracers (i.e., a cyclotron) must be located on-site at every PET facility. Second, because researchers can produce only a few images per day for each subject, statistical analyses combining data from several (i.e., usually at least six) subjects are necessary to generate reliable results. Images produced from a group of subjects, however, have considerably lower resolution than images produced from a single subject, because human brain anatomy varies slightly from person to person.

### Functional Magnetic Resonance Imaging

fMRI is the newest functional imaging technique. Like normal MRI, this approach does not use radioactive tracers, but exposes the subject to radio waves in the presence of a strong magnetic field. This treatment causes the nuclei of certain atoms in the brain to emit signals that can be detected by a scanner. The most commonly used form of fMRI analyzes the distribution of hemoglobin, the molecule in red blood cells that helps transport oxygen to the tissues. Specifically, this approach determines the ratio of hemoglobin that is still carrying oxygen (i.e., oxygenated hemoglobin) to hemoglobin that has already delivered its oxygen to the tissues (i.e., deoxygenated hemoglobin).

This fMRI approach is based on the fact that oxygenated and deoxygenated hemoglobin differ slightly in their magnetic properties, resulting in the emission of a signal that can be detected by special scanners. These scanners are so sensitive that they can detect signals from a volume (i.e., voxel) of brain tissue as small as a few millimeters on each side. The signal, which is proportional to the ratio of the concentration of oxygenated to deoxygenated hemoglobin inside the voxel, is called the blood oxygen level-dependent (BOLD) signal.

When a particular brain region is being used, the small arteries that supply the region with oxygen and other nutrients expand. As a result, the amount of oxygenated hemoglobin flowing into that region increases. In fact, the amount of oxygenated hemoglobin flowing into the tissue far exceeds the amount of deoxygenated hemoglobin produced by any increased metabolism in the active brain tissue. Thus, the ratio of oxygenated to deoxygenated hemoglobin—and, therefore, the BOLD signal—increases in the activated tissue, a change that can be measured by fMRI.

fMRI offers many advantages over earlier functional imaging techniques. First, fMRI does not expose subjects to radioactive substances, allowing researchers to study each subject on multiple occasions. Second, investigators can collect numerous images during a single imaging session, thereby allowing statistical analysis of data obtained from a single subject. This ability to analyze data from individual subjects means that the investigator can avoid the reduction in accuracy that occurs when brain images from many subjects are combined. Third, fMRI measurements represent brain activity during only a few seconds, enabling researchers to detect rapid changes in brain activity (i.e., enhancing temporal resolution). (PET and SPECT cannot measure brain activity during such a short interval.) Finally, the spatial resolution of fMRI exceeds that of the other modalities.

## A Preliminary Model of the Brain Regions Involved in Craving

Modell and colleagues (1990) developed the first model of neural systems that may play a role in alcoholism and particularly in craving. The model is based on the observation that the emotions associated with craving (as determined by patients’ self-reports) are similar to the compulsions described by patients with obsessive-compulsive disorder (OCD). Because OCD appears to be associated with dysfunction in a brain area called the orbital cortex, Modell and colleagues (1990) argued that a similar dysfunction could underlie craving for alcohol. Specifically, the investigators postulated a dysfunction in a type of brain circuit called a striatal-thalamocortical loop. The particular circuit proposed to be affected connects the orbital cortex, ventral striatum (including the nucleus accumbens and portions of the medial caudate nucleus), and thalamus. (For a list of the functions of these and other brain regions mentioned in this article, see table 1 below. For the locations of those regions, see the figure on p. 191.) Failure of this circuit may cause poor control of urges to drink. Striatal-thalamocortical loops are found in all terrestrial vertebrates (Reiner et al. 1998). In higher mammals, these circuits augment activity in small regions of the cortex that are involved in cognition, emotion, memory, or some specific actions, simultaneously inhibiting other nearby cortical regions. Striatal-thalamocortical loops receive nerve signals (i.e., input) from a set of cortical regions with related functions. This input serves to monitor the current behavioral context (i.e., ongoing behavior, motivation, and memories of similar circumstances) and provides the basis for selecting which small cortical regions, or circuits, need to be activated. In the human brain, multiple variants of these circuits affect different regions of the cortex and function in parallel (Alexander et al. 1986). The particular circuit proposed to be relevant to alcohol craving involves the orbital cortex.

The orbital cortex is part of the limbic system, the brain system that is thought to deal with emotions. One of the orbital cortex’s functions is to evaluate the motivational significance of stimuli that predict reward (Tremblay and Schultz 1999). In addition, the orbital cortex inhibits actions that are inappropriate for the person’s current situation (i.e., context-inappropriate actions). Damage to the orbital cortex can lead to antisocial behavior. Thus, it is reasonable to expect that a circuit serving this region may be involved in craving.

## Craving Studies in Alcoholics

Although several imaging studies have examined the effects of craving among cocaine abusers, only one such study has successfully induced and assessed craving among alcoholics (Modell and Mountz 1995). In that study, the investigators used SPECT technology to examine changes in cerebral blood flow in nine alcoholics^2^ who had ingested either water or a low dose of alcohol (0.03 grams per kilogram of body weight [g/kg]). The alcohol dose was too small to produce a direct pharmacological effect, but the researchers hypothesized that the dose would induce a desire to drink more alcohol. The subjects, who knew that they were receiving either water or alcohol but did not know which one they were receiving on a given day, always received the water on the first day of scans and alcohol on the second day of scans. Self-ratings obtained following the SPECT scans confirmed that the low alcohol dose did, in fact, induce a desire to drink in most subjects.

**Figure:**
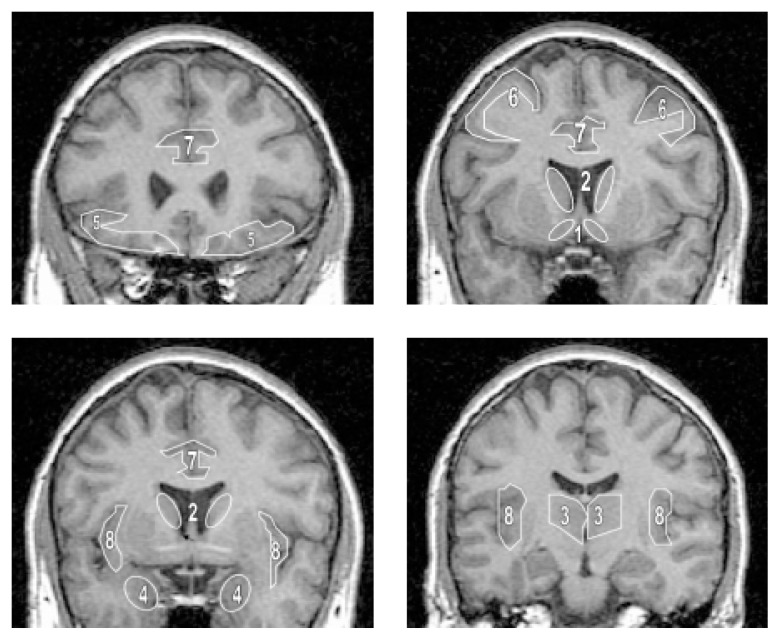
Locations of anatomical brain regions associated with craving as shown on a series of four MRI cross-sections taken from the top of the head (i.e., coronal slices). The images were generated using a T–1-weighted magnetic resonance scan of a normal human brain. This type of scan shows the brain as it usually appears in fresh or fixed post-mortem sections, with white matter appearing white, gray matter appearing gray, and the fluid surrounding the brain (i.e., the cerebrospinal fluid) appearing dark. NOTE: Numbers indicate the following brain regions: 1 = nucleus accumbens; 2 = caudate nucleus; 3 = thalamus; 4 = amygdalas; 5 = orbital prefrontal cortex; 6 = dorsolateral prefrontal cortex; 7 = anterior cingulate cortex; 8 = insular cortex. For a description of the function of these brain regions, see table 1. MRI = magnetic resonance imaging.

To facilitate the analysis, the investigators conducted a region-of-interest (ROI) analysis in which they measured changes in blood flow only in a few brain regions that they expected to be activated during craving. Those ROIs, which included the right and left caudate nucleus, the thalamus, and the orbital frontal cortex, were chosen based on the neural systems model of alcoholism discussed in the previous sections of this article. Of these regions, only the right caudate nucleus showed a significant increase in blood flow (and thus brain activity) during the craving condition. In addition, the extent of the subjects’ self-reported desire for alcohol predicted the magnitude of the increase in caudate blood flow following alcohol administration.

Modell and Mountz (1995) interpreted these results as confirming Modell and colleagues’ (1990) neural systems model of alcoholism. The lack of activation in the other components of the proposed circuit (i.e., the thalamus and the orbital cortex), however, weakens that interpretation. Furthermore, the results are suggestive at best, because the study included no nonalcoholic control subjects. Accordingly, it is uncertain whether the increased caudate activation resulted from alcohol administration itself or from the craving elicited by alcohol. The use of control subjects would have enabled researchers to distinguish between the two interpretations; the control subjects presumably would have exhibited similar blood flow changes if these changes had resulted from alcohol’s pharmacological effects but would not have experienced changes resulting from craving. Despite these limitations, however, the correlation between craving score and magnitude of blood flow change suggests that the activation of the right caudate nucleus may be related to craving.

Only one other published imaging study has analyzed brain regions potentially associated with craving in alcoholics. In that study, Hommer and colleagues (1997) attempted to induce craving for alcohol by injecting 18 hospitalized alcoholics^3^ and 12 control subjects with m-chlorophenylpiperazine (mCPP), an agent that has been used to induce craving among alcoholics. Widely used in psychiatric research, mCPP has been a probe for the activity of neuronal systems that use the neurotransmitter serotonin to relay signals from one neuron to the next. For this signal transmission, serotonin released by the signal-emitting neuron must interact with docking molecules (i.e., receptors) on the surface of the signal-receiving neuron. Several different serotonin receptors exist. The agent mCPP primarily mimics serotonin’s effects at one particular serotonin receptor (i.e., the 5HT_2C_ receptor) while blocking serotonin’s effects at other receptors. Alcohol administration also results in serotonin release and activation of the 5HT_2C_ receptor.

To identify brain regions involved in craving, Hommer and colleagues (1997) determined changes in glucose metabolism following injection of mCPP or an inactive saline solution (i.e., a placebo) using FDG PET. In all cases the placebo was always administered first, followed by mCPP 60 minutes later. Although other studies have found that mCPP can elicit both a desire to drink and subjective effects similar to alcohol intoxication among alcoholics, none of the alcoholics in this study (who were in the PET scanner when being assessed) expressed any increase in desire to drink or experienced any alcohol-like effects. In fact, neither alcoholics nor control subjects reported any significant subjective effects following mCPP administration other than a mild increase in anxiety. Both groups, however, exhibited increases in brain glucose metabolism. Among the control subjects, the increases occurred in the following brain regions:

- Two ridges (i.e., gyri) on the surface of the orbital cortex (i.e., the right medial and posterior orbital gyrus)
- The cerebellar^4^ hemispheres on both sides of the brain (i.e., bilaterally)
- The left nucleus accumbens
- The head of the caudate nucleus bilaterally
- Two groups of neurons located bilaterally in the thalamus (i.e., the anterior and medial dorsal nuclei of the thalamus)
- The dorsolateral prefrontal cortex
- The left insular cortex
- A gyrus on the temporal cortex located at the side of the brain (i.e., the left middle temporal gyrus)
- A gyrus on the cingulate cortex (i.e., the posterior cingulate gyrus).

Among the alcoholic subjects, mCPP significantly increased brain glucose metabolism in larger areas of the cerebellum and posterior cingulate gyrus than it did in the nonalcoholics. Conversely, the alcoholics showed a smaller area of mCPP-induced activation in the thalamus compared with the healthy volunteers, almost no activation in the orbital cortices, and no activation in the head of the caudate nucleus and the dorsolateral prefrontal cortex.

These results indicate that among nonalcoholics, treatment with a substance that induces or mimics serotonin release (i.e., a serotonergic challenge) activates striatal-thalamic circuits involving the orbital and dorsolateral prefrontal cortices and that these responses are reduced, or blunted, among alcoholics. Unfortunately, the relevance of these findings for identifying brain regions involved in craving remains unclear. The brain regions activated by mCPP in nonalcoholics include the parts of the striatum, thalamus, and cortex that have been implicated in mediating craving for alcohol by Modell and colleagues (1990). Conversely, alcoholics showed a blunted response to mCPP in these regions. The findings could indicate that the difficulties alcoholics experience with craving and with controlling their alcohol intake are associated with lower-than-normal activity in this circuit. In the absence of studies demonstrating a link between subjective craving and the function of striato-thalamic and orbitofrontal circuits, however, this model for the functional neuroanatomy of craving remains speculative.

## Craving Studies in Cocaine Abusers

In contrast to the few imaging studies conducted on alcohol craving, six studies have investigated craving for cocaine. Three of those studies used drug-related cues to elicit craving for cocaine, and three studies used drug administration (one study used cocaine itself and two studies used the chemical methylphenidate [Ritalin^®^]). Grant and colleagues (1996) used FDG PET to compare brain glucose metabolism in 13 non-treatment-seeking cocaine abusers and 5 control subjects. The two scanning sessions, separated by 1 week, took place while the subject viewed videotapes of objects associated with cocaine use or objects used in crafts. An ROI analysis demonstrated that among the control subjects, no significant differences in brain glucose metabolism existed in response to the two sets of cues. Among the cocaine abusers, however, brain metabolism increased significantly in response to the cocaine-related cues in several regions of the cortex, including the medial orbital, dorsolateral prefrontal, and medial temporal (including the amygdala) cortices as well as other cortical regions.^5^ No significant changes in brain metabolism occurred in the basal ganglia or thalamus. In addition, the subjects’ self-ratings of craving showed a significant positive correlation with metabolism in the dorsolateral prefrontal cortex and the medial temporal cortex.

Recently Childress and colleagues (1999) reported the results of an ^15^O PET study of cocaine craving that included 14 treatment-seeking cocaine abusers and 6 non-cocaine-abusing control subjects. The investigators performed six sequential scans on each subject, both during rest periods and while the subjects were watching cocaine-related and non-cocaine-related videotapes. Using ROI analysis the study demonstrated that in the cocaine abusers, but not in the control subjects, cocaine craving elicited by the videotapes resulted in increased blood flow in the amygdala and anterior cingulate cortex and decreased blood flow in the caudate nucleus and in the globus pallidus, which connects the striatum and the thalamus.

The videotapes that Childress and colleagues (1999) used to elicit cocaine craving also were used in an fMRI study of six cocaine abusers (whose treatment status was not specified) and six control subjects (Maas et al. 1998). The study was designed to determine whether the changes observed in the two previously described studies (Childress et al. 1999; Grant et al. 1996) also could be demonstrated using fMRI. To this end the investigators used a ROI analysis of 10 brain regions that had been identified in the other two studies as being responsive to cocaine craving. Among those regions, however, only the anterior cingulate gyrus and the left dorsolateral prefrontal cortices were activated in the cocaine abusers, and only the results for the anterior cingulate gyrus reached statistical significance.

Another fMRI study of cocaine craving used cocaine administration to induce both euphoria and craving in 10 non-treatment-seeking cocaine abusers (Breiter et al. 1997). The study included no control subjects. Cocaine administration induced BOLD-signal increases in a large number of limbic and other brain regions, whereas a saline solution caused only small changes in the BOLD signals measured in the lateral prefrontal and temporo-occipital cortices. The largest increases following cocaine administration occurred in the region of the nucleus accumbens, caudate nucleus, thalamus, and anterior cingulate and insular cortices, as well as in a few other brain regions.^6^ In the amygdala the BOLD signal decreased in response to cocaine administration.

The researchers took advantage of the fact that fMRI allows the collection of many images during a brief period of time and examined the relationship between subjective response and the time course of brain activation. They found that the activity of the brain regions that showed an early peak response followed by a rapid return to pre-cocaine levels correlated with euphoria. Conversely, the activity of the regions that showed a sustained increase in activity (i.e., the nucleus accumbens/subcallosal gyrus region and the basal forebrain) correlated positively with craving. Finally, the time course of the BOLD signal in the amygdala negatively correlated with craving.

Two studies used methylphenidate to elicit craving in cocaine abusers who had been hospitalized for treatment for at least 3 weeks. In one of those studies, changes in brain glucose metabolism were measured in 20 cocaine abusers before and after intravenous administration of methylphenidate (Volkow et al. 1999). ROI analysis demonstrated that methylphenidate increased brain glucose metabolism in the anterior cingulate gyrus, right thalamus, and cerebellum. Furthermore, methlyphenidate-induced increases in brain glucose metabolism in the right orbital frontal cortex and right caudate nucleus correlated positively with craving for cocaine. Because the study included no control subjects, however, it is unclear whether this pattern of activation is unique to cocaine abusers or would also occur among non-drug users.

The other study involving methylphenidate examined changes in the release of the neurotransmitter dopamine using PET technology and an agent called ^11^C-raclopride, which can interact with a specific receptor for dopamine (i.e., the D2 receptor) (Volkow et al. 1997). Dopamine release may be associated with the pleasurable or rewarding effects of AODs and with the motivation to use AODs to experience those rewarding effects. Methylphenidate causes the release of dopamine in the brain, which then displaces ^11^C-raclopride bound to D2 receptors. Thus, changes in ^11^C-raclopride binding provide a measure of how much dopamine is released in response to methylphenidate administration. The study found that compared with control subjects, cocaine abusers exhibited decreased dopamine release in the striatum, specifically the caudate nucleus, but increased release in the thalamus. Furthermore, the magnitude of thalamic dopamine release among the cocaine abusers was positively correlated with craving for cocaine. However, the relevance of this correlation is unclear, because both the release of dopamine and the concentration of dopamine receptors are low in the thalamus.

### Comparison of the Study Results

The six imaging studies conducted among cocaine abusers and the one successful study conducted in alcoholics identified numerous brain regions that appear to be involved in craving (for a summary, see table 2, p. 194; also see the figure on page 191 for the locations of those brain regions). The specific pattern of brain regions that is activated appears to depend to some extent on the technique used to elicit craving. One group of studies induced craving by administering small amounts of the abused drug itself or of an agent with similar effects. Those studies reported increased activity in the thalamus during craving. In contrast, studies that used visual cues (i.e., videotapes) to induce craving reported increased brain activity primarily in the dorsolateral prefrontal cortex.

The prefrontal cortex generally is activated during tasks involving both working memory (i.e., the short-term storage of information) and episodic memory (i.e., the recall of sequences of past events). Accordingly, activation of the dorsolateral prefrontal cortex during cue-induced craving may occur because craving based on visual cues requires the activation of certain memory circuits, whereas such memories may not be required for the occurrence of drug-induced craving. It is unclear why cue-induced craving fails to activate the thalamus, because both drug-induced and cue-induced craving are associated with activity changes in the anterior cingulate gyrus, caudate nucleus, amygdala, and orbital cortices. All these regions, along with the thalamus, are components of various basal ganglia-thalamocortical circuits; because of their anatomical connections, one would expect these regions to be functionally linked (Alexander et al. 1986).

Although the studies discussed in this article indicate that the brain regions mentioned earlier are activated during craving, one cannot exclude the possibility that other regions are activated as well. All but two of the studies used ROI methods of image analysis and therefore examined only limited portions of the brain. In fact, most of the studies limited their analysis to the caudate nucleus; thalamus; amygdala; and orbital, dorsolateral frontal, and anterior cingulate cortices. The only two studies to analyze data from the entire brain (Breiter et al. 1997; Hommer et al. 1997) detected activation in the nucleus accumbens and insular cortex in addition to the regions listed above. (The results of the study by Hommer and colleagues are not summarized in table 2, because the subjects in that study did not report craving during the scanning procedure.) The nucleus accumbens and insular cortex, as well as the other activated regions, are components of striatal-thalamocortical circuits. Although the precise function of those circuits in humans is unknown, they are thought to be involved in emotion, motivation, and social behavior (Alexander et al. 1986). In addition, the insular cortex is involved in visceral functions related to feeding and emotion.

## A Revised Model of the Functional Neuroanatomy of Craving

The brain regions identified as being involved in craving for alcohol or cocaine all participate in various basal ganglia-thalamocortical circuits, which influence a person’s sequential choice of behaviors (e.g., actions, thoughts, and emotions) based on the behavioral context (see table 1). Three of those regions (i.e., the caudate nucleus, thalamus, and orbital frontal cortex) were included in the initial craving model developed by Modell and colleagues (1990). In addition, the dorsolateral prefrontal cortex may be involved in craving, as discussed earlier in this article. The anterior cingulate cortex, which was activated in most studies of craving, is involved in the regulation of attention and emotion (Posner and Rothbart 1998) and may be particularly important in learning associations between certain stimuli and rewards (Bussey et al. 1997). Finally, the amygdala is commonly associated with fear but also relays information about stimuli associated with reward to the orbital cortex (Hatfield et al. 1996).

The brain regions associated with craving also are generally associated with motivation (Koob et al. 1998), a state that can be described as inducing those behaviors that are related to satisfying an organism’s needs. Furthermore, with the exception of the thalamus, all these brain regions receive information from dopamine-releasing neurons that are located in the midbrain but extend their axons to these regions and release dopamine there. Dopamine is often considered a reward neurotransmitter whose release provides the drive behind behavior undertaken in anticipation of a pleasurable outcome (i.e., appetitively motivated behavior). Conversely, reductions in dopamine activity may be involved in motivating behavior undertaken in order to avoid the loss of pleasure (i.e., aversively motivated behavior) (Schultz 1998). This bivalent nature of dopamine’s motivational function may mirror the mixture of motivations that appear to comprise craving. On the one hand, craving can result from an alcoholic’s desire for the pleasurable effects of alcohol (i.e., an appetitive drive). On the other hand, craving also can result from an alcoholic’s desire to avoid the unpleasant experiences of withdrawal or another negative emotional state (i.e., an aversive drive). Thus, craving may involve either aversive or appetitive motivation and, at times, perhaps both simultaneously.

Studies of the activities of individual dopamine neurons in the monkey brain in response to certain stimuli (Schultz 1998) have helped generate a framework for understanding the neurophysiology of craving and may provide suggestions for the design of future imaging studies. In those studies, animals repeatedly were exposed to arbitrary stimuli (i.e., colored lights) that predicted the opportunity to gain a reward. When the stimulus initially was paired with a reward, it rapidly elicited a burst of activity in the dopamine-releasing neurons of the midbrain. Thus, it appears that dopamine-releasing neurons provide a signal indicating the presence of an important new association to be learned between a stimulus and a subsequent reward. With continued pairings of the stimulus and the reward, however, the dopamine-releasing neurons stop responding, indicating that those neurons do not react to stimuli with a well-established, consistent relationship to a reward. In other words, once an association is learned, no further dopamine activity is required to maintain that connection. In fact, if dopamine activity indicates that the organism must learn a new association between a stimulus, a behavior, and a reward, then responses of dopamine-releasing neurons in familiar, unchanging situations might even be counterproductive.

Both alcohol and cocaine increase dopamine concentrations throughout the various basal ganglia-thalamocortical circuits that have been implicated in craving in the studies reviewed in this article. Consequently, both drugs should put a person’s brain into a state similar to the state that occurs when a person makes the initial connection between a stimulus or behavior and a desired reward. Such a state may be both pleasant and arousing and therefore may encourage further drug consumption (i.e., is reinforcing). In addition, the elevated dopamine concentrations should prime the person to associate the stimuli preceding alcohol or cocaine use (e.g., certain objects, places, people, thoughts, or feelings) with the pleasure experienced when the drug is taken. Thus, when those stimuli are encountered again, they should initially elicit a burst of activity in the dopamine-releasing neurons, thereby leading to anticipation of the reward and AOD-seeking behavior. This scenario corresponds to appetitively motivated craving.

What happens, however, when an alcoholic encounters stimuli associated with alcohol use in a situation in which he or she cannot drink? The experiments in monkeys mentioned earlier also demonstrated that when a stimulus which previously had been paired with a reward stopped predicting that reward, appearance of that stimulus soon began to elicit a decrease in dopamine activity (Schultz 1998). This observation leads to two questions. First, if a burst of dopamine neuron activity signals the organism to learn an association, does a decrease in dopamine neuron activity indicate that a connection must be “unlearned”? Second, if a burst of dopamine neuron activity leads to a pleasant or arousing state, does a decrease in dopamine neuron activity lead to a dysphoric state, perhaps akin to frustration? Although the answers to those questions are unknown, the questions themselves suggest that the model of craving underlying the design of the studies reviewed here is too simplistic.

To date, all functional imaging studies have treated craving as a state characterized by only one pattern of behaviors or emotions (i.e., a unitary state). The studies by Schultz (1998) and the resulting questions, however, indicate that the desire to drink elicited by a cue or low alcohol dose in a situation in which the subject knows that more alcohol is available likely differs from the desire to drink elicited in a situation in which further alcohol consumption is impossible. Although subjects in one of the studies reviewed here were allowed to use cocaine after the cue exposure and scanning were completed (Grant et al. 1996), no study has systematically examined the effect of the perceived availability of the drug on brain states associated with craving.

## The Future of Functional Imaging of Craving

Although the imaging studies discussed in this article have begun to shed light on the brain regions involved in craving, future studies should address several additional issues. First, researchers should design studies that can distinguish the effects of appetitive and aversive motivation. For example, experiments could compare the effects of craving on brain activity under conditions in which the subject knows that AODs will or will not be available.

Second, imaging studies in healthy people without AOD addiction should attempt to identify patterns of brain activity generally associated with appetitive and aversive motivation (i.e., not associated with AODs). The results of such studies could be compared with the patterns of brain activation found during craving for AODs, thereby enabling researchers to understand craving in the context of how the brain deals with motivation in general.

Third, although this review has focused on dopamine, researchers know that many other neurotransmitters are involved in motivational processes. Thus, the neurotransmitters gamma-aminobutyric acid (GABA), glutamate, and opiate peptides—and, perhaps, serotonin—all help mediate the reinforcing properties of alcohol (Koob et al. 1998). Once the nature of the brain states associated with craving has been characterized in more detail, it may be possible to explore systematically how drugs that selectively affect specific neurotransmitters can modify those brain states.

In summary, the concept of craving, which has its origin in popular psychology, may not correspond to unitary brain states observable with functional imaging techniques. By carefully designing and analyzing functional imaging studies, however, researchers may be able to determine whether it would be more useful for researchers to divide the process of craving into two or more components. If functional imaging is to be more than colored pictures that humans use to convince themselves that psychological constructs have a physical reality in the brain, researchers must be able to use this approach to test hypotheses and change their models of how the mind works.

## Figures and Tables

**Table 1:** Various Brain Regions Involved in Craving and Their Main Functions

Brain Region	Function
**Subcortex**	
*Striatal thalamocortical loop*	Connects functionally related areas of the cortex with the striatum, which projects to the thalamus via the globus pallidus; in the thalamus, information is combined with information from the related cortical areas and then sent back to one region of the frontal cortex Functions to select one output (e.g., emotion, thought, or motor action) from among several competing potential responses based on behavioral context
Nucleus accumbens (ventral striatum) (1)	Connected to limbic systems and orbital prefrontal cortex; related to emotion and motivation
Caudate nucleus (dorsal striatum) (2)	Connected to medial and lateral prefrontal cortex; related to cognition and motivation
Thalamus (3)	Relays information between cortical regions and other brain areas, including the striatum
Amygdala (4)	Learning of stimuli predicting reward and punishment; fear response
**Cortex**	
*Prefrontal cortex*	Working memory; planning and motor preparation; inhibition of context-inappropriate behavior
Orbital (5)	Olfaction, behavioral inhibition, and evaluation of stimuli’s motivational significance
Dorsolateral (6)	Spatial working memory and cognition
Anterior cingulate (7)	Attention and motivation
**Insular Cortex** (8)	Visceral function, olfaction, taste, and emotion

NOTE: The numbers in parentheses refer to the locations of the brain regions in the accompanying four-part figure in this article (see page 191).

**Table 2:** Changes in Activity in Several Brain Regions Under Conditions of Craving for Alcohol or Cocaine as Determined in Studies Using Various Funtional Imaging Techniques

Imaging Technique Used	Nucleus Accumbens	Caudate Nucleus	Thalamus	Amygdala	Orbital Cortex	Dorsolateral Frontal Cortex	Anterior Cingulate Cortex	Insular Cortex
**Alcohol**								
SPECT (Modell and Mountz 1995)		Right only ⇧+ correlation						
**Cocaine**								
FDG PET (Grant et al. 1996)				⇧ + correlation	⇧	⇧ + correlation	⇧	
^15^O PET (Childress et al. 1999)		⇩		⇧		⇧	⇧	
fMRI (Maas et al. 1998)					⇧	Left only + correlation	⇧ + correlation	
fMRI (Breiter et al. 1997)	⇧ + correlation	⇧	⇧	⇩ – correlation		⇧	⇧	⇧
FDG PET (Volkow et al. 1999)		Right only + correlation	Right only	Right only	+ correlation	Right only		
^11^C-raclopride PET (Volkow et al. 1999)		⇩	⇧ + correlation					

NOTE: Arrows represent increases and decreases in activity; “+ correlation” and “– correlation” refer to a positive or negative correlation, respectively, between the degree of brain activity and the extent of craving; the designations “right” and “left” refer to structures located in the right and left hemispheres of the brain, respectively. SPECT = single photon emission computed tomography; FDG = fluorodeoxyglucose; PET = positron emission tomography; fMRI = functional magnetic resonance imaging; ^15^O = radioactively labeled oxygen;^11^C = radioactively labeled carbon. Raclopride is an agent that interacts with a receptor for the neurotransmitter dopamine.

## Footnotes

1 For definitions of this and other technical terms used in this article, see the glossary, p. 196.

2 All study participants were treated for alcoholism on an outpatient basis and had been abstinent for at least 1 week.

3 All of these subjects had been abstinent for at least 3 weeks.

4 The cerebellum is a brain region located at the lower back of the brain that is primarily involved in motor functions.

5 These additional cortical regions include the retro-splenial, temporo-parietal, and peri-striate cortices.

6 These additional areas include the subcallosal gyrus, putamen, and basal forebrain region.

## GLOSSARY

Axon: Part of the nerve cell (i.e., neuron) that extends to other neurons and transmits information to those cells.
Basal ganglia: Groups of nerve cells located above the thalamus in the white matter of the brain. These nerve cells, which include the caudate nucleus, putamen, and pallidum, help control motor and cognitive activity.
Cerebral: Pertaining to the cerebrum, the largest and uppermost section of the brain, which is divided into the right and left hemispheres.
Half-life: The time required for one-half of a given amount of a radioactive compound to decay.
Neurotransmitter: A chemical messenger used by nerve cells to relay signals from one cell to the next; common neurotransmitters include dopamine and serotonin.
Photon: The smallest quantity of electromagnetic energy. This energy may occur in the form of X-rays, gamma rays, or a quantum of light.
Resolution: The ability of an imaging technique to distinguish between two adjacent structures; greater resolution indicates better image quality.
Tracer: A radioactive molecule used in imaging techniques that enables the investigator to visualize a biological process.
